# Public views of acceptability of perinatal mental health screening and treatment preference: a population based survey

**DOI:** 10.1186/1471-2393-14-67

**Published:** 2014-02-12

**Authors:** Dawn Kingston, Sheila McDonald, Suzanne Tough, Marie-Paule Austin, Kathy Hegadoren, Gerri Lasiuk

**Affiliations:** 1 University of Alberta, 11405-87th Avenue, Edmonton, T6G 1C9 Alberta, Canada; 2 University of Calgary, Calgary, Canada; 3 University of New South Wales, Sydney, Australia

**Keywords:** Perinatal mental health, Screening, Acceptability, Treatment, Postpartum, Antenatal, Depression, Anxiety, Stress, Mental health literacy

## Abstract

**Background:**

At a prevalence rate of 13-25%, mental health problems are among the most common morbidities of the prenatal and postnatal periods. They have been associated with increased risk of preterm birth and low birthweight, child developmental delay, and poor child mental health. However, very few pregnant and postpartum women proactively seek help or engage in treatment and less than 15% receive needed mental healthcare. While system-related barriers limit accessibility and availability of mental health services, personal barriers, such as views of mental health and its treatment, are also cited as significant deterrents of obtaining mental healthcare. The purposes of this population-based study were to identify the public’s views regarding mental health screening and treatment in pregnant and postpartum women, and to determine factors associated with those views.

**Methods:**

A computer-assisted telephone survey was conducted by the Population Research Laboratory with a random sample of adults in Alberta, Canada. Questions were drawn from the Perinatal Depression Monitor, an Australian population-based survey on perinatal mental health; additional questions were developed and tested to reflect the Canadian context. Interviews were conducted in English and were less than 30 minutes in duration. Descriptive and multivariable regression analyses were conducted.

**Results:**

Among the 1207 respondents, 74.8% had post-secondary education, 16.3% were 18-34 years old, and two-thirds (66.1%) did not have children <18 years living at home. The majority of respondents strongly agreed/agreed that all women should be screened in the prenatal (63.0%) and postpartum periods (72.7%). Respondents reported that when seeking help and support their first choice would be a family doctor. Preferred treatments were talking to a doctor or midwife and counseling. Knowledge of perinatal mental health was the main factor associated with different treatment preferences.

**Conclusions:**

The high acceptability of universal perinatal mental health screening among the public provides a strong message regarding the public value for routine screening during pregnancy and postpartum periods. Perinatal mental health literacy is the most prominent determinant of screening and treatment acceptability and preference. Efforts to enhance population literacy as part of a multifaceted perinatal mental health strategy may optimize pregnant and postpartum women’s mental health.

## Background

With a prevalence of 15 to 25% [1,2], depression, stress and anxiety are the most common complications experienced by pregnant and postpartum women. Without treatment, up to 48% of women with prenatal anxiety and 70% of those with prenatal depression [3] continue to experience symptoms through the postpartum period [4,5] and into their children’s early years of life [6,7]. Based on two decades of well-conducted longitudinal studies, the evidence is clear that even mild to moderate perinatal distress can have serious adverse effects on mothers and children, including increased risk of preterm birth and low birthweight [8], child developmental delay [9-11], impaired mother-child bonding [12], and poor child mental health [13,14].

However, very few pregnant and postpartum women proactively seek help or engage in treatment [15,16] and less than 15% of pregnant and postpartum women receive needed mental healthcare [17]. While system-related barriers limit accessibility and availability of mental health services, personal barriers such as views of mental health and its treatment are also cited as significant deterrents of obtaining mental healthcare [16].

The Theory of Planned Behavior is a salient framework for understanding the role that views play in explaining the disparity observed in rates of pregnant and postpartum women who require and seek mental healthcare. Empirical testing of the Theory of Planned Behavior (TPB) suggests that both personal (e.g., normative) and societal beliefs play an important role in behavior [18]. Based on this theory, behavior is driven by an individual’s personal beliefs (formed from his/her attitude toward a behavior and desired consequences), the subjective norm (e.g., societal beliefs) related to performing the behavior, and perceived behavioral control (i.e., the perceived ease with which the behavior can be accomplished) [18]. Important referents in an individual’s life (e.g., spouse, friend, family) may play a key role in the formation of his/her normative beliefs. Collectively, normative beliefs form the subjective norm – the perceived obligation that an individual feels about engaging in a particular behavior [18]. Within this framework, a pregnant or postpartum woman’s views and intentions regarding mental healthcare are shaped by her perception of significant others’ views as well as prevailing societal views, and these in turn influence her actions regarding screening and treatment.

Studies have demonstrated that the decision-making processes among childbearing families are heavily informed by the experiences and opinions of friends and family members [16,19]. Treatment preferences and decisions are also highly influenced by societal beliefs about the causes of poor mental health and the stigma related to treatment, which impacts help-seeking behaviors, outcomes and adherence to treatment [20-22]. Indeed, the stigma associated with having a perinatal mental health problem and receiving treatment is identified as a major deterrent to women’s help-seeking and treatment [16]. While the inverse association between stigma (e.g., negative stereotyping) and help-seeking is well-established in the general population [23], it may be more pronounced during the perinatal period as women experience shame and guilt for not feeling happy and content during pregnancy or after delivery. The stigma of being an incompetent mother or a danger to their children adds to their substantial burden of concern [24-26].

Given the impact of societal beliefs about mental health on acceptability, preferences, and uptake of treatment [20,23,27], identifying the public’s normative views regarding perinatal mental health screening and treatment is instrumental in understanding childbearing women’s views and behaviors, and how to normalize help-seeking behavior for women and families. It is also the basis on which to design effective messages that counter myths and misperceptions, enhance public understanding, and attain public support for needed policy and practice changes.

Few studies have explored the public’s views of perinatal mental health [28], and none have done so in Canada. The purposes of this study were to identify the public’s views regarding mental health screening and treatment in pregnant and postpartum women, and to determine factors associated with those views.

## Methods

### Survey instrument

The Alberta Survey B is a cross-sectional survey conducted annually by the Population Research Laboratory (PRL) at the University of Alberta (Canada). The survey content varies yearly as different sponsors submit questions of interest. In 2012, the Alberta Centre for Child, Family and Community Research sponsored 15 items designed to gather information on perinatal mental health literacy in the province. In Alberta, mental health screening and treatment are not components of routine prenatal care in the majority of primary care settings. Postnatally, all women are to be assessed with the Edinburgh Postnatal Depression Scale and given appropriate referrals by a public health nurse at their infant’s 2-month immunization visit; however, this practice is not consistently implemented across the province. Therefore, questions addressed topics related to knowledge of the effects of anxiety and depression occurring during pre-conception, prenatal, and postpartum periods; help-seeking for prenatal and postpartum anxiety and depression; and screening and treatment for pregnant and postpartum women with anxiety or depression. Questions were drawn from the Australian *Perinatal Depression Monitor,* a 26-item survey designed to measure population-based awareness, attitudes, and knowledge regarding prenatal and postnatal mental health. This survey was developed through extensive consultation with health professionals and consumers and was patterned after other population-based Australian surveys used in the past decade to assess mental health literacy [28]. Additional questions were developed and tested for the Alberta Survey B to address the Canadian context. The latter were evaluated for face and content validity by several researchers, clinicians, and policy-makers with expertise in perinatal mental health. All questions were pre-tested by the PRL on 20 households to check wording, response categories, question order, interviewer instructions, and length of interview [29].

### Survey procedures

Random-digit dialing of several provincial telephone banks generated a random sample of households from rural and urban areas and ensured that the sampling frame included households with and without telephone directory listings [29]. Respondents were eligible to participate if they were: 1) ≥18 years; and 2) contacted by direct dialing. The survey was designed to recruit equal numbers of male (n = 603) and female (n = 604) respondents with two-thirds living in urban centers. The study protocol was approved by the Research Ethics Board at the University of Alberta. Oral consent was obtained from each participant by the interviewer after the interviewer read a standard statement regarding the voluntary nature of his/her participation and confidentiality of the information collected in accordance with the Alberta Freedom of Information and Protection of Privacy Act.

Data were collected between June 5, 2012 and June 27, 2012 by trained PRL interviewers using computer-assisted telephone interviews. Interviews were conducted in English and each lasted a maximum of 30 minutes. A random sample of 10% of the respondents were followed-up by PRL supervisors to verify accuracy of data collection.

### Outcomes

The main outcomes were the public’s views related to: 1) universal prenatal mental health screening; 2) universal postnatal mental health screening; 3) help-seeking; and 4) treatment for perinatal mental health problems (i.e., accurate knowledge of treatment options; preferred treatment options) (Table 1). Prenatal and postnatal mental health were defined as anxiety, depression, or stress occurring in the prenatal and postnatal periods, respectively.

**Table 1:** Definitions of outcomes

**Acceptability of prenatal and postnatal screening**	Views of prenatal screening were considered to be acceptable if participants responded to the item *All women should be screened for depression and anxiety in pregnancy* with a response of *strongly agree* or *agree* (versus *strongly disagree, disagree*, or *neither agree nor disagree*). A similar item related to postnatal screening assessed its acceptability.
**Help-seeking**	To assess the first source of help and support, we asked respondents to identify *who* would be their first choice if they or their partner had depression during pregnancy or after delivery (e.g., partner, friend, family doctor, obstetrician, midwife, public health nurse, parent, other relative).
**Treatment for perinatal mental health problems: accurate knowledge of treatment options**	Knowledge of perinatal mental health treatment options was considered accurate if participants responded to the item *The only way of treating anxiety and/or depression is with medication* with a response of *strongly disagree* or *disagree* (versus *strongly agree, agree*, or *neither agree nor disagree*). Preferred treatment options were assessed by the single item, *If you had depression during pregnancy or after you had a baby, what kind of treatment options would you prefer*, where respondents had opportunity to select all that applied from a list (see Tables 5 and 6).

### Definitions

High level of prenatal mental health knowledge was defined by a response of *strongly* or *somewhat agree* to the item, *Women who have had anxiety or depression in the past (before they became pregnant) are more likely to experience anxiety or depression when they are pregnant* and/or *Women who have anxiety or depression during pregnancy are more likely to experience postpartum depression*, while those with low prenatal knowledge answered *strongly disagree, somewhat disagree,* or *neither agree nor disagree* to one or more of these items*.* High postnatal knowledge was defined by a response of *strongly* or *somewhat agree* to at least one of three questions, including, *Women who have postpartum depression find it more difficult to respond to their baby’s cues* and/or *Women who have postpartum depression find it more difficult to respond to the needs of their partner and other children* and/or *Partners of women who have postpartum depression are also at risk for depression*, whereas those with low postnatal knowledge responded *strongly disagree, somewhat disagree,* or *neither agree nor disagree* to one or more of these questions. High perinatal knowledge was defined as having high prenatal and/or postnatal knowledge; conversely, low perinatal knowledge was defined as having low prenatal and/or postnatal knowledge.

Consistent with findings of the Maternity Experiences Survey, childbearing age was defined as the lowest age of inclusion in the survey (18 years) to 34 years [19]. Because previous surveys of mental health literacy have demonstrated that attitudes toward mental health are influenced by personal experience [22], we asked participants to identify whether they knew someone who had had anxiety or depression after having a baby.

### Analysis

Descriptive data (N, %) were generated. Unadjusted odds ratios (UORs) and 95% confidence intervals (CIs) were calculated for each independent variable and outcome. Variables associated with the outcomes at a level of p < 0.10 were considered for inclusion in the multivariable models. Multivariable logistic regression models were constructed with all variables entered simultaneously to generate adjusted odds ratios (AORs) and 95% CIs with p < 0.05 defining statistically significant factors. Only those factors that were significant at p < 0.05 are presented in the final, multivariable models. Model robustness was assessed by entering excluded variables back into the final models. The analysis was conducted using SPSS (Version 21.0.0).

## Results

### Study participants

In total, 1207 completed interviews were conducted. Of the total eligible participants (n = 10,563), 3029 refused, 29 interviews were incomplete (i.e., the respondent opted to stop the interview prior to completion), 102 interviews involved language problems, 4559 were not available, and 1637 were not contacted (but estimated as eligible). The overall response rate was 27.6% (calculated as completed interviews/completed interviews + refusals + incompletes + language problems) [29]. Item non-response rate was low (<4.0%). Respondents were most commonly 45-64 years of age (45.8%), with 16.3% (n = 192) being 18-34 years old (Table 2). Two-thirds of households had no children under 18 years of age living in the household (66.3%). The majority of respondents were married or living common-law (68.6%), had completed post-secondary education (74.7%), were born in Canada (81.8%) and were Caucasian (85.4%) (Table 2). Over half of the respondents reported knowing a woman who had experienced postpartum anxiety or depression (57.4%) (Table 3). More than two thirds of respondents had high knowledge levels regarding prenatal mental health and over 85% had high levels of postnatal mental health knowledge (Table 3).

**Table 2:** Socio-demographic characteristics of respondents of the 2012 Alberta Survey B (Alberta, Canada) (N = 1209)

**Demographic characteristics**	**N (%)**
**Sex**	
Male (planned^**¶**^)	603 (50.0)
Female	604 (50.0)
**Employment status**	
Unemployed	467 (38.7)
Employed	739 (61.3)
**Had children <18 years living in household**	
No	798 (66.3)
Yes	406 (33.7)
**Age (years)**	
18-24	53 (4.5)
25-34	139 (11.8)
35-44	211 (17.9)
45-54	269 (22.9)
55-64	269 (22.9)
65+	235 (20.0)
**Marital status**	
Single/widowed/divorced	377 (31.4)
Married/common-law	824 (68.6)
**Education (highest level completed)**	
Less than high school	96 (7.9)
High school complete	209 (17.4)
Post-secondary	898 (74.7)
**Born in Canada**	
No	220 (18.2)
Yes	987 (81.8)
**Born in Alberta (Canada)**	
No	403 (40.9)
Yes	584 (59.1)
**Ethnicity**	
Non-Caucasian	186 (14.6)
Caucasian	1023 (85.4)
**Income (annual household income)**	
<$40,000	129 (13.7)
≥$40,000	815 (86.3)
**Residence**	
Rural/other	401 (33.3)
Urban	804 (66.7)

Note. Some variables do not total 1209 due to missing responses.

^**¶**^The survey was designed to sample equal proportions of males and females.

**Table 3:** Summary of participant responses to perinatal mental health items in the 2012 Alberta Survey B (Alberta, Canada) (N = 1209)

**Responses to perinatal mental health items**	**N (%)**
**Knowledge of prenatal mental health**	
High	831 (70.5)
Low	347 (29.5)
**Knowledge of postnatal mental health**	
High	1028 (87.4)
Low	148 (12.6)
**Personal experience with someone who experienced postpartum anxiety or depression**	
Yes	678 (57.4)
No	503 (42.6)
**All women should be screened for depression and anxiety in pregnancy**	
Strongly agree or agree	760 (63.0)
Strongly disagree or disagree	195 (16.1)
Neither agree nor disagree	158 (13.1)
Don’t know	57 (4.7)
No response	37 (3.1)
**All women should be screened for depression and anxiety after they have a baby**	
Strongly agree or agree	878 (72.7)
Strongly disagree or disagree	131 (10.9)
Neither agree nor disagree	113 (9.4)
Don’t know	50 (4.1)
No response	35 (2.9)
**The only way of treating anxiety and/or depression is with medication**	
Strongly agree or agree	67 (5.6)
Strongly disagree or disagree	895 (74.2)
Neither agree nor disagree	143 (11.8)
Don’t know	69 (5.7)
No response	33 (2.7)
**First choice for help and support**	
Family doctor	470 (38.9)
Partner	213 (17.7)
Mother or father	114 (9.4)
Friend	110 (9.1)
Psychologist	49 (4.1)
Obstetrician or midwife	58 (4.8)
Public health nurse	44 (3.6)
Mental health therapist	37 (3.1)
Other relative	25 (2.1)
Clergy/spiritual leader	25 (2.1)
Don’t know	19 (1.6)
No response	44 (3.6)

Note. Some variables do not total 1209 due to missing responses. Some totals may exceed 100% due to rounding.

### Views on prenatal and postnatal screening

The majority of respondents strongly agreed/agreed that all women should be checked for depression and anxiety during pregnancy (63.0%) and after having a baby (72.7%) (Table 3). Among women of childbearing age, 79.2% strongly agreed/agreed that prenatal screening should be routinely conducted, and 88.5% responded similarly for postnatal screening – proportions that were significantly higher than those observed in the total sample (data not in table). In the multivariable analysis examining factors associated with acceptability of prenatal and postnatal screening (Table 4), women were more likely than men to strongly agree/agree that all women should be screened pre- and postnatally, as were those with high prenatal and postnatal knowledge. In addition, Caucasian respondents were less likely to strongly agree/agree that women should be screened prenatally, while those who had personal experience with a woman with postpartum depression/anxiety were more likely to agree that all women should be screened postnatally.

**Table 4:** **Factors associated with public acceptability of prenatal and postnatal screening, accurate knowledge of treatment options, and seeking a healthcare provider as the first choice for help in Alberta, Canada (UOR, AOR, 95**% **CI)**

**Independent variable**	**Prenatal screening**			**Postnatal screening**			**Accurate knowledge of treatment options**			**Healthcare provider first choice for help**		
	**n (%)**	**UOR (95% CI)**	**AOR (95% CI)**	**n (%)**	**UOR (95% CI)**	**AOR (95% CI)**	**n (%)**	**UOR (95% CI)**	**AOR (95% CI)**	**n (%)**	**UOR (95% CI)**	**AOR (95% CI)**
Age												
18-34 years	129 (69.4)	1.28 (0.91, 1.80)		143 (76.9)	1.14 (0.79, 1.65)		158 (84.9)	*1.95 (1.27, 2.99)	1.96 (1.26, 3.05)	82 (44.3)	*0.52 (0.38, 0.72)	0.54 (0.38, 0.75)
≥35 years	610 (63.9)	1.00		713 (74.5)	1.00		713 (74.3)	1.00		561 (60.3)	1.00	
Sex												
Female	413 (69.5)	*1.52 (1.19, 1.93)	1.43 (1.11, 1.84)	486 (81.7)	*2.12 (1.61, 2.78)	1.91 (1.44, 2.54)	451 (75.7)	0.93 (0.71, 1.22)		273 (46.3)	*0.38 (0.30, 0.49)	0.38 (0.30, 0.50)
Male	345 (60.1)	1.00		390 (67.8)	1.00		443 (76.9)	1.00		383 (69.3)	1.00	
Employment												
Employed	478 (66.4)	1.19 (0.93, 1.52)		539 (74.8)	0.99 (0.75, 1.30)		582 (80.6)	*1.83 (1.39, 2.40)	1.71 (1.29, 2.28)	379 (54.1)	*0.69 (0.54, 0.88)	0.60 (0.46, 0.78)
Unemployed	279 (62.4)	1.00		336 (75.0)	1.00		312 (69.5)	1.00		277 (63.0)	1.00	
Marital status												
Married/ common-law	525 (65.2)	1.03 (0.79, 1.34)		610 (75.7)	1.14 (0.86, 1.51)		621 (77.0)	1.15 (0.86, 1.53)		456 (57.6)	1.01 (0.78, 1.31)	
Single/widowed/divorced	231 (64.5)	1.00		263 (73.3)	1.00		268 (74.4)	1.00		199 (57.3)	1.00	
Has post-secondary education												
Yes	573 (65.6)	1.12 (0.85, 1.48)		658 (75.2)	1.06 (0.78, 1.43)		695 (79.2)	*1.83 (1.36, 2.46)		491 (57.5)	1.01 (0.77, 1.33)	
No	182 (63.0)	1.00		215 (74.1)	1.00		195 (67.5)	1.00		162 (57.2)	1.00	
Income (annual household)												
≥$40,000	518 (64.8)	0.78 (0.52, 1.18)		601 (75.1)	1.05 (0.68, 1.62)		633 (79.0)	*1.73 (1.14, 2.62)		442 (56.9)	1.00 (0.69, 1.47)	
<$40,000	87 (70.2)	1.00		92 (74.2)	1.00		85 (68.5)	1.00		71 (56.8)	1.00	
Residence												
Urban	509 (65.2)	1.04 (0.80, 1.34)		588 (75.2)	1.05 (0.80, 1.39)		613 (78.2)	*1.37 (1.03, 1.81)		430 (56.4)	0.87 (0.68, 1.12)	
Rural/other	249 (64.3)	1.00		288 (74.2)	1.00		281 (72.4)	1.00		226 (59.6)	1.00	
Has children <18 yr in household												
Yes	269 (67.4)	1.19 (0.92, 1.53)		304 (76.2)	1.11 (0.84, 1.47)		322 (80.5)	*1.45 (1.08, 1.95)		207 (52.5)	*0.74 (0.58, 0.94)	
No	487 (63.6)			570 (74.2)			569 (74.0)			447 (60.0)		
Born in Canada												
Yes	614 (64.2)	0.85 (0.62, 1.16)		712 (74.3)	0.85 (0.60, 1.21)		738 (77.0)	1.22 (0.87, 1.71)		548 (58.7)	*1.33 (0.99, 1.80)	
No	144 (67.9)	1.00		164 (77.4)	1.00		156 (73.2)	1.00		108 (51.7)	1.00	
Born in Alberta												
Yes	356 (63.5)	0.92 (0.71, 1.21)		421 (74.8)	1.06 (0.79, 1.42)		433 (76.9)	0.99 (0.73, 1.35)		317 (57.6)	0.90 (0.69, 1.17)	
No	258 (65.3)	1.00		291 (73.7)	1.00		305 (77.0)	1.00		231 (60.3)	1.00	
Ethnicity												
Caucasian	631 (63.7)	*0.73 (0.51, 1.04)	0.65 (0.45, 0.94)	738 (74.3)	0.86 (0.59, 1.27)		771 (77.6)	*1.55 (1.09, 2.22)	1.49 (1.02, 2.19)	571 (59.0)	*1.53 (1.10, 2.12)	1.61 (1.13, 2.29)
Non-caucasian	120 (70.6)	1.00		131 (77.1)	1.00		118 (69.0)	1.00		81 (48.5)	1.00	
Prenatal knowledge												
High	577 (69.9)	*2.10 (1.62, 2.72)	1.72 (1.31, 2.27)	656 (79.3)	*2.15 (1.63, 2.84)	1.62 (1.20, 2.19)	647 (78.1)	*1.39 (1.04, 1.86)		464 (56.8)	0.94 (0.72, 1.22)	
Low	177 (52.5)	1.00		216 (64.1)	1.00		244 (72.0)	1.00		186 (58.3)	1.00	
Postnatal knowledge												
High	700 (68.5)	*3.36 (2.35, 4.80)	3.03 (2.07, 4.44)	804 (78.5)	*3.81 (2.66, 5.45)	2.91 (1.98, 4.27)	815 (79.5)	*3.24 (2.26, 4.65)	2.78 (1.89, 4.09)	584 (57.5)	1.02 (0.70, 1.49)	
Low	57 (39.3)	1.00		71 (49.0)	1.00		79 (54.5)	1.00		70 (56.9)	1.00	
Personal experience												
Yes	457 (67.8)	*1.31 (1.03, 1.68)		537 (79.6)	*1.73 (1.32, 2.26)	1.37 (1.03,1.82)	541 (79.9)	*1.54 (1.17, 2.02)	1.38 (1.03, 1.84)	357 (53.7)	*0.69 (0.54, 0.88)	0.75 (0.58, 0.97)
No	300 (61.6)	1.00		338 (69.3)	1.00		352 (72.1)	1.00		298 (62.7)	1.00	

Note. UOR-Unadjusted odds ratio; AOR-Adjusted odds ratio; CI-Confidence interval. Some variables do not total 1209 due to missing responses.

*Meets criteria for inclusion in multivariable models (p <0.10). Variables in the multivariable model are adjusted for all other variables in the model. Multivariable models present only those variables significant at p < 0.05.

### Views on help-seeking

Respondents most commonly identified the family doctor (38.9%) or the partner (17.7%) as the first choice of help and support if they (women respondents) or their partner (male respondents) suffered with depression during pregnancy or after the birth of the baby (Table 3). Few respondents indicated they would seek help from friends (9.1%) or parents (9.4%), and less than 5% would go to either an obstetrician/midwife, public health nurse, psychologist, mental health therapist, or clergy/spiritual leader as their first option (Table 3). A multivariable analysis was performed to determine the independent factors associated with choosing a healthcare provider as the first source of help and support compared to support from other sources (e.g., friends, family). Caucasian respondents were more likely to indicate that they would choose a healthcare provider as their first choice for help (versus non-Caucasian) (Table 4). Respondents who were female, in childbearing years, employed, and who had personal experience in knowing a woman with postpartum anxiety/depression were less likely to visit a healthcare provider as their initial source of help and support (Table 4).

### Views on Treatment

Most respondents (74.2%) correctly disagreed that medication was the only form of treatment for anxiety/depression (Table 3). In the multivariable analysis, significant, independent factors associated with accurate knowledge of treatment options were being 18-34 years of age, being employed, being Caucasian, having high postnatal knowledge, and having personal experience in knowing a woman with postpartum anxiety/depression (Table 3).

The most common treatment preferences endorsed by respondents were talking to a doctor or midwife (81.6%) and counseling (79.8%) (Table 5). Less than half of respondents endorsed medications or internet/web-based self-help options. Factors associated with preference for individual treatment options are found in Table 6. The most consistent factor associated with treatment preferences was high perinatal knowledge. In other words, respondents with high knowledge of causes and consequences of perinatal mental health were nearly 2 to 5 times as likely to endorse all treatment options (after controlling for other factors) compared to those with low perinatal knowledge. Among the demographic factors, respondents who were employed were more likely to prefer peer support, help with parenting, internet/web-based self-help and dietary/nutritional approaches compared to those who were unemployed. Respondents with post-secondary education were more likely to endorse counseling, phone support, or nutritional approaches than those without post-secondary education. The only option associated with being born in Canada was phone support. Little difference was apparent by sex, with the exception that women preferred internet-based self-help and nutritional supplements. Medication was less preferred by respondents aged 18-34 years compared to those 35 years of age and older, but was endorsed as a treatment option by those with high perinatal knowledge and with personal experience.

**Table 5:** Perinatal mental health treatment preferences reported in the 2012 Alberta Survey B (Alberta, Canada) (n,%)

**Variable**	**Counselling**	**Peer support**	**Phone support**	**Medication**	**Diet/nutritional supplements**	**Parenting help**	**Talk to doctor or midwife**	**Internet/web based self-help**
	**n (%)**	**n (%)**	**n (%)**	**n (%)**	**n (%)**	**n (%)**	**n (%)**	**n (%)**
18-34 years	154 (80.2)	149 (77.6)	92 (47.9)	69 (35.9)*	125 (65.1)	140 (72.9)	160 (83.3)	77 (40.1)
Female	497 (82.6)*	434 (72.1)	318 (52.8)	263 (43.7)	417 (69.3)*	436 (72.4)*	492 (81.7)	252 (41.9)*
Married/common-law	671 (81.5)*	614 (74.6)	441 (53.6)	363 (44.1)	533 (64.8)*	583 (70.8)	683 (83.0)*	318 (38.6)
Has post-secondary education	738 (82.3)*	663 (73.9)	492 (54.8)*	398 (44.4)*	588 (65.6)*	648 (72.2)*	740 (82.5)	351 (39.1)*
Household income ≥ $40,000/annum	665 (81.8)*	617 (75.9)	450 (554)	357 (43.9)	517 (63.6)	587 (72.2)*	675 (83.0)*	309 (38.0)
Employed	604 (81.8)*	562 (76.2)*	410 (55.6)*	322 (43.6)	495 (67.1)*	545 (73.8)*	607 (82.2)	306 (41.5)*
Urban residence	648 (80.6)	598 (74.4)	420 (52.2)	336 (41.8)	503 (62.6)	568 (70.6)	651 (81.0)	318 (39.6)*
Has children < 18 yr in household	333 (82.2)	307 (75.8)	218 (53.8)	173 (42.7)	274 (67.7)*	299 (73.8)*	337 (83.2)	173 (42.7)*
Born in Canada	786 (79.8)	716 (72.7)	535 (54.3)*	418 (42.4)	635 (64.5)*	698 (70.9)	804 (81.6)	375 (38.1)
Born in Alberta	460 (78.9)	427 (73.2)	313 (53.7)	255 (43.7)	375 (64.3)	411 (70.5)	471 (80.8)	230 (39.5)
Caucasian	817 (80.0)	751 (73.6)	546 (53.5)	446 (43.7)	655 (64.2)	722 (70.7)	835 (81.8)	389 (38.1)
High perinatal knowledge	915 (84.0)*	837 (76.9)*	613 (56.3)*	493 (45.3)*	726 (66.7)*	809 (74.3)*	933 (85.7)*	433 (39.8)*
Personal experience	388 (77.1)*	358 (71.2)*	246 (48.9)*	180 (35.8)*	288 (57.3)*	340 (67.6)*	399 (79.3)*	175 (34.8)*
Total sample	964 (79.8)	883 (73.2)	639 (52.9)	515 (42.7)	762 (63.2)	848 (70.3)	985 (81.6)	454 (37.6)

Note. Some variables do not total 1209 due to missing responses.

*Variables are associated with the outcome in unadjusted logistic regression analyses at p < 0.10 and therefore meet criteria for entry to multivariable models.

**Table 6:** **Factors associated with treatment preferences reported in the 2012 Alberta Survey B (Alberta, Canada) (AOR, 95**% **CI)**

**Independent variable**	**Counselling**	**Peer support**	**Phone support**	**Medication**	**Diet/nutritional supplement**	**Help with parenting**	**Talk with a doctor or midwife**	**Internet/web-based self-help**
	**AOR (95% CI)**	**AOR (95% CI)**	**AOR (95% CI)**	**AOR (95% CI)**	**AOR (95% CI)**	**AOR (95% CI)**	**AOR (95% CI)**	**AOR (95% CI)**
18-34 years	-	-	-	0.70 (0.51,0.97)	-	-	-	-
Female	-	-	-	-	1.70 (1.33,2.19)	-	-	1.42 (1.11,1.80)
Employed	-	1.40 (1.07,1.83)	-	-	1.58 (1.22,2.04)	1.49 (1.15,1.94)	-	1.57 (1.22,2.02)
Has post-secondary education	1.70 (1.23,2.36)	-	1.38 (1.05,1.80)	-	1.39 (1.05,1.84)	-	-	-
Married/common-law	-	-	-	-	-	-	-	-
Has children <18 yr in home	-	-	-	-	-	-	-	-
Household income ≥ $40,000/annum	-	-	-	-	-	-	-	-
Born in Canada	-	-	1.37 (1.01,1.86)	-	-	-	-	-
High perinatal knowledge	4.75 (3.00,7.51)	3.10 (1.98,4.84)	3.34 (2.03,5.48)	1.92 (1.13,3.24)	3.02 (1.90,4.80)	3.92 (2.49,6.16)	4.52 (2.85,7.15)	1.89 (1.14,3.16)
Personal experience	-	-	-	1.55 (1.21,1.98)	-	-	-	-

Note. AOR-Adjusted odds ratio; CI-Confidence interval; ‘-‘ variable not significant in final model; Variables in the multivariable model are adjusted for all other variables in the model. Significance level is p < 0.05.

## Discussion

To our knowledge, this is the first population-based study to describe the general public’s views regarding perinatal mental health screening and adds to the limited knowledge regarding public views of help-seeking and treatment. Key findings indicate that acceptability of routine prenatal and postnatal mental health screening is high, the person of choice for help and support is the family physician, the most preferred treatment options are talking to a doctor or midwife and counseling, and knowledge of perinatal mental health is the main factor associated with treatment preference.

### Views on prenatal and postnatal screening

Although recommended by the American College of Obstetricians and Gynecologists [30] and other national organizations [31,32], perinatal mental health screening is not a component of routine prenatal or postnatal care in 80% of North American settings [33-35]. It is noteworthy, then, that the findings demonstrate high acceptability of routine perinatal mental health screening– particularly among women of childbearing age where 79% and 89% agreed that all women should be screened prenatally and postnatally, respectively (data not shown). This is an important observation within the context of a healthcare system that does not actively promote nor accommodate routine perinatal mental health screening – and highlights the public’s recognition of the value of universal screening. While some studies describe women’s barriers to perinatal mental health screening [36,37], and others report healthcare providers’ beliefs that women are reluctant to be screened [25], the findings of this study do not support concerns that universal screening is unacceptable.

The rates of acceptability of prenatal and postnatal screening that we observed are lower than those reported in Australia’s national perinatal mental health literacy survey of public views (78% and 83%, respectively) [28] and Gemmill et al.’s study where 97% of Australian postpartum women agreed that all new mothers should be screened for depression [38]. This difference may in part be due to the implementation of Australia’s National Perinatal Depression Initiative in 2008 - a federal initiative that supports universal prenatal and postnatal mental health screening and treatment access through funding, resources, education, and enhanced community awareness [32]. The finding that individuals who are knowledgeable about the causes and consequences of prenatal and postnatal depression/anxiety were more likely to endorse universal screening provides evidence of the link between knowledge and mental health screening and is consistent with the impact of health literacy on other forms of screening [39].

### Views on help-seeking

In this study, respondents who were female, 18-34 years of age, or employed were more likely to identify family and friends as their first choice for help (rather than a healthcare professional). While family and friends can be supportive influences, some studies have reported that families and friends deter women from help-seeking if they normalize symptoms that require attention or exhibit limited understanding of perinatal mental health [16,25,40]. Although both family physicians and obstetricians provide the majority of perinatal care within the Canadian healthcare system, the finding that 38.9% of respondents endorsed family physicians as the first point of contact while less than 5% identified obstetricians as such may reflect the public’s belief that mental healthcare is outside of obstetricians’ scope of practice [41,42]. This belief may be influenced in part by the lack of routine screening in obstetrical settings, the limited time allotted per visit, or the perception that obstetricians are not qualified to provide mental healthcare [42]. Pregnant and postpartum women have also cited the fear of being prescribed medications as the only treatment option and having their concerns dismissed as significant barriers to seeking help from a perinatal provider [43]. The impact of personally knowing someone with depression or anxiety on viewing medication favorably as a treatment option has been reported previously in population-based surveys of mental health [22].

Very few respondents indicated that they would seek a public health nurse as the first choice for help, despite the early contact made by public health nurses through home visitation in the early postpartum period. However, family physicians, obstetricians, and nurses are key points of access to mental healthcare, particularly since women receive care from these providers fourteen times on average across the perinatal period [19]. Indeed, Bennett et al. reported that 78% of pregnant women were willing to seek mental healthcare from their obstetrician after he/she initiated discussion about depression at some point during care [42]. There is a need to enhance the public’s understanding of the role that these practitioners play in obtaining or accessing mental healthcare.

An interesting finding was that only 17.7% of survey respondents identified the partner as the first choice for help. Although no studies have reported this previously, it may reflect a fear of being viewed as an incompetent mother, a concern that the partner would dismiss the symptoms as normal [40], or a desire not to burden the partner.

#### ***Views on treatment***

The preferences for face-to-face treatment approaches involving counseling and support were similar to the population-based Australian study [28] and those reported in pregnant women [44]. The findings of this study extend existing research by providing insight into the determinants of treatment options. Notably, respondents who were knowledgeable about the causes and consequences of poor perinatal mental health were more likely to endorse a wide variety of treatment options. This is consistent with qualitative studies in which postpartum women identified the need for a “menu” of treatment choices and the ability to switch between options as necessary [24]. Although health literacy is only one factor that impacts help-seeking behavior [39,45,46], our study demonstrated that it was the most prominent influence driving respondents’ acceptability of treatments. The association between having ‘high’ levels of knowledge of perinatal mental health and finding all forms of treatment acceptable (Table 5) may in part be explained by our definition of ‘high’ perinatal mental health knowledge (i.e., accurate knowledge of causes and consequences of poor perinatal mental health). In other words, respondents who understood the nature of perinatal mental health problems recognized the benefit of both non-pharmacologic and pharmacologic treatment approaches. The association between knowledge of perinatal mental health and acceptability of pharmacologic therapy is important to note in that women’s low acceptability of pharmacologic therapy during the perinatal period can be a significant barrier to improvement in women for whom medication may be beneficial [16,25].

Although internet-based/self-help therapy was of the least endorsed treatment options, it is worth noting that women (versus men) and those who were employed (versus unemployed) were more likely to prefer this form of treatment. Goodman et al. found that 25.5% of pregnant women rated internet-based/self-help approaches as among the top three treatment choices for depression [44]. As an easily accessible, highly effective treatment option [47,48], internet-based/self-help therapy addresses some of the deterrents that pregnant and postpartum women describe for not obtaining help for anxiety and depression, including preferring to deal with it themselves, being too busy, not having childcare, and not being comfortable or being too embarrassed to talk to someone else [49,50]. Indeed, Reay et al. reported that 93% of women with perinatal depression and anxiety who engaged in self-help perceived it as helpful, compared to 73% who found counseling helpful [50].

Accurate knowledge regarding the range of treatment options and their effectiveness is a central concept of health literacy, and one that is understudied in perinatal mental health. As anticipated, findings from this study parallel others that demonstrate that being Caucasian, being younger, and knowing someone with a mental health problem are associated with higher mental health literacy, including knowledge of treatment options [51]. This study adds to this body of knowledge by identifying the main factor associated with accurate treatment knowledge as being knowledgeable about postnatal mental health. Thus, this finding is consistent with our other observations related to the positive impact of perinatal mental health knowledge on screening and treatment acceptability.

The results were similar to other studies that report the public’s [28] and postpartum women’s [16] preferences for counseling over pharmacological therapies. It is interesting, however, that while obstetricians and family physicians view their perceived lack of expertise as a barrier to engaging in perinatal mental healthcare [43,52,53], the public views them as the main source of treatment. Thus, this study elucidates a disconnect that exists between healthcare provider assumptions and public expectations regarding perinatal mental healthcare – a key point that should dispel concerns surrounding the role that they play in screening, referral, and treatment [43,53]. In addition, the findings provide insight into the broader realm of public decision-making where talking to physicians remains the preferred form of treatment.

### Limitations

The limitations of this study must be acknowledged. The low response rate of 27.6% raises the risk of selection bias in the sample. However, the sample characteristics (employment; marital status; education; income) reflect the demographic composition of the province with the exception that the mean age of our sample (M = 42.7 years; median = 45 years) was slightly higher than that of the province (M = 36.5) [54]. Given the inverse association between age and accurate knowledge of treatment that we observed, our rates of mental health literacy may be lower than those in the province overall. The response rate was similar to those achieved in the Alberta Survey in past years and is greater than the Australian survey on perinatal mental health (13%) [51]. However, the low response rate is consistent with those reported in other population-based studies examining public attitudes toward mental health [22,23,28]. Similarly, the age distribution that we observed among respondents (i.e., 42.9% ≥55 years) is comparable to that reported in the Australian study of perinatal mental health (i.e., 39.5% ≥55 years) [28]. As the questions were a component of a larger survey of diverse topics, we were limited in the scope of topics we could address and could not gather data on other important areas such as the public’s views of causes of perinatal depression/anxiety [9,55]. Participants were not asked to identify their occupation, and thus we were unable to isolate the views of healthcare professionals. Finally, study participation was limited to those with telephone access.

## Conclusions

The high acceptability of universal perinatal mental screening among the public and particularly women of childbearing age provides a strong message to clinicians and policy-makers that merits an actionable response. Little progress in the implementation of universal screening has been accomplished in Canada and the U.S. since the recommendations for routine psychosocial screening were proposed by the American College of Obstetricians and Gynecologists in 2006 [30]. While a main deterrent cited by clinicians is women’s lack of acceptability of perinatal mental health screening [25], these findings did not support that concern. Given that perinatal mental health literacy was the primary factor associated with the acceptability of treatment, providing psycho-education (i.e., education surrounding mental health disorders and treatment) that enhances awareness of mental health across the perinatal period should represent a component of an effective strategy for improving treatment rates [25].

## Abbreviations

PRL: Population Research Laboratory; UOR: Unadjusted odds ratio; AOR: Adjusted odds ratio; CI: Confidence interval.

## Competing interests

The authors declare that they have no competing interests.

## Authors’ contributions

All authors listed have contributed sufficiently to the project to be included as authors. DK conceptualized the study, participated in the development of the survey, was a consultant to the Population Research Laboratory on the testing of the survey, conducted data analysis, interpreted findings, drafted the manuscript and made final revisions. SM contributed to conceptualization of the study, conducted data analysis, reviewed the findings, interpreted the data, critically reviewed the manuscript and provided final approval of the submitted version. MPA reviewed and interpreted the results, critically reviewed the manuscript and approved the final version to be published. ST contributed to conceptualization of the study, participated in the development of the survey, interpreted the findings, critically reviewed the manuscript and approved the final version to be published. KH aided in the development of the survey, critically reviewed the manuscript and approved the final version to be published. GL contributed to survey development, critically reviewed the manuscript and approved the final version for publication. All authors read and approved the final manuscript.

## Pre-publication history

The pre-publication history for this paper can be accessed here:

http://www.biomedcentral.com/1471-2393/14/67/prepub
